# Client satisfaction on community based health insurance scheme and associated factors at Boru Meda Hospital, Northeast, Ethiopia: institutional based cross-sectional study

**DOI:** 10.1186/s12913-021-07223-4

**Published:** 2021-11-30

**Authors:** Mulugeta Tasew Hailie, Seid Legesse Hassen, Minwuyelet Maru Temesgen

**Affiliations:** 1 Amhara Public Health Institute, Planning, Monitoring, and Evaluation Directorate, Dessie, Ethiopia; 2 Amhara Public Health Institute, Research and Technology Transfer Directorate, Dessie, Ethiopia

**Keywords:** Community-based health insurance, Client satisfaction, Hospital Ethiopia

## Abstract

**Background:**

Community-based health insurance systems are usually voluntary and characterized by community members pooling funds and protecting themselves against the high costs of seeking medical care and treatment for illness. Client satisfaction with health service provision during the implementation of health insurance schemes has often been neglected. This study aimed to determine client satisfaction with the community-based health insurance scheme and associated factors.

**Methods:**

An institutional-based cross-sectional study design was applied from February 22–March 11 /2019. A total of 420 study participants were included in the study using a systematic random sampling technique. Data were collected using a pretested semi-structured interviewer-administered questionnaire with a patient exit interview. Bivariate and multivariate logistic regression analyses were used to identify factors associated with Community-based Health Insurance of client satisfaction. Statistical significance was decided at a *p*-value less than 0.05.

**Result:**

A total of 420 community-based health insurance clients of health service users participated in the study with a 100% response rate. The overall client satisfaction was 80% at 95% Cl (76.1, 83.9), respondents who have perceived that partially or none availability of prescribing drugs were 0.09 times less likely satisfied as compared to full availability of prescribing drugs (AOR =0.09; 95% Cl: (0.04, 0.19)). Besides, study participants waiting time to consult service providers within 30 min were more satisfied than those who were delayed 60 min and above (AOR =3.16; 95% Cl: (1.19, 8.41)).

**Conclusion:**

Community-based health insurance client satisfaction provided in the present study was 80% indicating low proportion. Full availability of prescribing drugs, clients renewed their community-based health insurance membership, and preference of clients to use the hospital for future health care need were positively associated with client satisfaction while the perception of waiting time before physician consultation negatively affected client’s satisfaction. Therefore, the hospital management members and service providers need to give attention to reduce waiting time preceding consultation, improve drug availability, and sustain the hospital preference by the client.

## Background

Community-based health insurance (CBHI) schemes are usually voluntary and characterized by community members pooling funds and to protect themselves against the high costs of seeking medical care and treatment for illness [1, 2]. Inability to pay the out-of-pocket (OOP) expenditure has been pushed as one of the main impediments to access healthcare particularly for the poor and the vulnerable population [3]. Over the last twenty years, CBHI has rapidly grown as a health financing tool in low and middle-income countries [4]. It has become one of the key risk-protection schemes and is expected to play a great role in helping the country move towards universal coverage in the health sector [5, 6].

However, globally every year around 150 million people suffer from financial devastation and about 100 million are pushed into poverty because of high out-of-pocket payments for health care services. The majority of these people reside in developing countries [7–9]. Health insurance schemes in many low and middle-income countries (LMICs), most especially in the African continent are still in their early stages of implementation with the goal of Universal Coverage [10]. Direct payment for seeking care is considered as regressive as it inhibits access to health services for the poor. It is also considered to contribute to the impoverishment of families due to having to pay for unexpected health care services at the time of illness [11].

Ethiopia has one of the worst health outcomes in the world. OOP payment at the time of seeking health care continues to be one of the major sources of financing for health in Ethiopia [12, 13]. However, in recent years a new health policy results from some improvements in the population’s health, and a new health financing strategy led to critical changes in the financing structure of healthcare [14]. The Federal Ministry of Health (FMOH) of Ethiopia developed a mutual health insurance strategy for CBHI schemes. The CBHI targets those employed in the rural and informal sectors [15]. The Government of Ethiopia launched CBHI schemes in 13 pilot woredas in Amhara, Oromia, Southern Nations, Nationalities, and Peoples (SNNP), and Tigray National Regional States in 2010/11 to provide risk protection for those employed in the rural and the informal sectors. The Ethiopian health insurance agency now is working by enhancing risk pooling between the rich and poor as well as between healthy and sick [16, 17]. The benefits packages of CBHI in Ethiopia include all family health services and curative care that are part of the essential health package which excludes dental implantation and optics services [16]. The client’s satisfaction is a multidimensional and broader concept taking into account the individual perceptions, expectations, and experience together [18, 19]. It is determined by service quality, clients’ expectations, subjective disconfirmation, and emotions experienced during service delivery [20–22]. The satisfaction of enrollees and its influencing factors have been providing evidence that has assisted in policy and decision making [23]. Furthermore, client satisfaction studies allow service users’ voices to be heard and confirm their experience for improved health care planning [24, 25]. The main reasons cited for dissatisfaction with the quality of care include drug stock out, lengthy waiting time, lack of courtesy on the part of the staff assigned in the facilities, and inadequate availability of diagnostic service [25]. These problems cause a major challenge to CBHI members, greater than to the general community; because members have to pay the out-of-pocket payment for drugs, diagnostic, and other health services in non-contracted health facilities [15]. It leads to additional expenses and causes CBHI scheme members dissatisfied. Therefore, conducting such research on this area is important to determine CBHI client satisfaction and its associated factors in the hospital setting to alleviate the problem and assured the continuous attractiveness of the care contracted.

In general client satisfaction studies are rarely collected or used as part of designing health insurance schemes in developing countries like Ghana about 76% [26], in Tamil Nadu, south India 82% [20], There are fewer studies from the Ethiopian scenario related to CBHI client satisfaction [27, 28], but the level of CBHI client satisfaction in the study area is not studied so far. Therefore, this study aimed to measure the level of institutional-based CBHI clients’ satisfaction after a visit to health care services and factors associated with it in Boru Meda hospital. So this study provides evidence-based information to improve health service delivery and also, help to fill gaps that ultimately contribute to the desirable quality of Clients’ services in the hospital and enhancing the level of clients’ satisfaction.

## Methods and materials

### Study design, period, and setting

An institutional-based cross-sectional study was carried out between February 22–March 11/2019, at Boru Meda Hospital, Northeast, Ethiopia. Boru Meda hospital is located 410 km away from Addis Ababa the capital city of Ethiopia and 10 km from Dessie city administration in the north direction. It is a primary hospital running an annual budget of Birr more than 20 million with a bed capacity of over 80 and a total staff exceeding 270. It provides services for approximately 1051 inpatients, 964 emergency cases, and 46,651outpatient attendants per year among these 9330 clients were enrolled in CBHI. Since the beginning of the CBHI pilot scheme implementation in Ethiopia, this hospital has been providing services for different woredas for insured clients who had taken contract agreement.

### Source population and study population

The study population included all CBHI members who newly joined and renewed CBHI membership at the time of the study period and those clients who got health care service in the hospital. Clients under 18 years, their parents, or caretakers were interviewed.

### Sample size and sampling procedure

The sample size was calculated using the single population proportion formula with the following assumptions; proportion of client satisfaction 54.4% from Wolaita Sodo university teaching hospital [29], using a 5% margin of error at 95% confidence level. The final sample size was 420 after considering 10% non-response rate. The total sample size was proportionately allocated for each service delivery unit in the hospital depending on the average number of clients who visited the service area one month before the start of the study. Then study participants were identified by systematic random sampling and coded with the help of triage order at the patient entry point. In addition, Simple random sampling was done for the first clients to get the starting point. Thereafter, depending on sampling interval clients’ coming to the service delivery exit were enrolled in the study until the required sample size was obtained.

### Data collection procedures

Data were collected using pre-tested structured questionnaires by exit interview in four confidential rooms. The questionnaire was adapted from the Ethiopia health insurance agency evaluation report 2015 [15] with a slight modification made with the objective of this particular study and to fit with the local context. The questionnaire, which was initially prepared in English and translated into the local language, Amharic, by those proficient in the language, and checked for consistency. Five percent of the questionnaires were pre-tested before the actual data collection period in a comparable setting. Finally, the interview was administered by six health care providers who have BSc nurse for data collection, and one health officer for supervision activities was recruited. Supervisors and principal investigators oversaw the data collection daily.

### Data processing and analysis

The collected data were checked for completeness and consistency, then coded, and entered into Epi Info 7.1, and exported to SPSS version 21 statistical software for final analysis. Principal Component Analysis with varimax rotation was used to compute the wealth index of the client. Categorical variables used measure income were transformed into separate dichotomous (0–1) indicators. These indicators and those that are continuous are then examined using a principal components analysis to produce a common factor score for each client’s household. The resulting combined wealth index has a mean of zero and a standard deviation of one. Once the index is computed, ranking each person in the population by his or her score, and then dividing the ranking into three equal categories (poor, medium, and rich), each comprising 33.3% of the client. Simple descriptive analyses were used to describe the study population correlated to relevant variables and presented using text, table, frequency, and percentage. Variables that have a *p*-value ≤of 0.2 on bivariate analyses were entered in the multivariate logistic regression model to identify independent variables of enrollment into CBHI. *P* < 0.05 was considered statistically significant. The strength of association and precision were examined using an adjusted odds ratio at a 95% confidence interval. The model fitness was checked by Hosmer and Lemeshow’s goodness of fit test.

### Operational definition

Clients need to receive medical care for their illness and/or caregivers for children, elders, and seriously ill families.

#### Level of client’s satisfaction

Five measuring items were used in the scale to measure satisfaction together yield a maximum score of 25 and a minimum score of 5. Satisfaction level was measured by the responses for every five items was summed and transformed to give an individual level satisfaction score from 0 to100% for each item used as a percentage mean score.

#### **Overall satisfaction level**

Seventy five percent and above response rate of the five satisfaction measuring items were categorized as “satisfied” and those who were satisfied in less than 75% of the five satisfaction measuring items were categorized as “Dissatisfied” [29].

#### **Wealth index**

The index was constructed using clients’ household asset data via a principal components analysis. Once the index is computed, ranking each person in the population by his or her score, and then dividing the ranking into three equal categories, each comprising 33.3% of the clients [30] .those whose score < 33.3 were categorized as poor, those score between 33.3 and 66.6 were categorized as medium and those score > 66.6 were categorized as rich.

## Results

### Socio-demographic characteristic of the respondent

A total of 420 CBHI members of health service users participated in the study with a 100% response rate. Of the total participants, 228 (54%) were male and 268 (63.8%) were married. The median age of respondents was 40 years with an Interquartile range of 33 (22–55) years (Table 1).

**Table 1:** Socio-Demographic characteristics of Respondents with health care services provided at Boru Meda hospital, Feb.22-Mar.11/ 2019 (*n* = 420)

Variable	Number	Percent
Age category (in year)		
< =18	**87**	**20.7**
19–29	**48**	**11.4**
30–39	**69**	**16.4**
40–49	**62**	**14.8**
50–59	**72**	**17.1**
> =60	**82**	**19.5**
Sex		
Male	**228**	**54.3**
Female	**192**	**45.7**
Educational status		
Unable to read and write	**230**	**54.8**
Able to read and write	**28**	**6.7**
1–8	**115**	**27.4**
9–12	**45**	**10.7**
TVET/Collage/University)	**2**	**0.5**
Religion		
Orthodox	**113**	**26.9**
Muslim	**307**	**73.1**
Marital status		
Married	**268**	**68.8**
Single	**119**	**28.3**
Widowed	**19**	**4.5**
divorced	**14**	**3.3**
Occupation		
Farmer	**299**	**71.2**
Unemployed	**23**	**5.5**
Student	**72**	**17.1**
Merchant	**8**	**1.9**
Others	**18**	**4.3**
Ethnics		
Amhara	**418**	**99.5**
Oromo	**2**	**0.5**
Fee waiver beneficiary before CBHI		
Yes	**36**	**8.6**
No	**384**	**91.4**
Wealth index		
Poor	**140**	**33.3**
Medium	**141**	**33.6**
Rich	**139**	**33.1**

### Institutional aspect and pattern of clients Visit

Among the total number of hospital visited CBHI clients 120 (28.1%) were due to fever followed by 97 (23.1%) dermatology cases. More than 177 (42.1%) of clients were waiting for 30–60 min to consult a physician. Out of 247 Clients who were sent to laboratory diagnosis 151 (61.4%) completed within 30–60 min. More than 76% of clients came far from 10 km and only 34.3% of clients have transport access.

### Client satisfaction and quality of service

This exit interview assessed the satisfaction of CBHI member clients during service delivery. Among the outpatient clients the cleanliness of the hospital was highest satisfaction rate 387 (98.7%) and satisfaction was rated lowest 299 (71.2%) with the availability of drugs. Out of 28 admitted Clients who participated in study 21(75%) of them were satisfied by the attentiveness and adequate follow-up of nursing staff. Out of 420 clients, 247 (58%) were ordered to laboratory diagnosis. Thirty-one (7.3%) clients had got radiology service. All clients got prescription paper for drugs and Supplies, but only 73.1% of them were getting all the prescribed drugs and 119 (28.3%) were dissatisfied concerning with the availability of drugs in the hospital (Table 2).

**Table 2:** Level of client’s satisfaction with different components of health care services at Boru Meda hospital, Feb.22-Mar 11/ 2019 (*n* = 420)

Characteristics	v.sat (%)	Sati (%)	Neut (%)	Dissat (%)	v.disat (%)
Overall quality of service	138 (32.9)	203 (48.3)	41 (9.8)	28 (6.7)	10 (2.4)
Availability of drugs	101 (24)	200 (47.6)	33 (7.9)	71 (16.9)	15 (3.6)
Availability of diagnostic facility (*n* = 247)	78 (31.6)	143 (57.9)	9 (3.6)	16 (6.5)	1 (0.4)
Cleanliness of facility	244 (58.1)	169 (40.2)	2 (0.5)	3 (0.7)	2 (0.5)
Short wait time until seen by physician	109 (26)	229 (54.5)	29 (6.9)	44 (10.5)	9 (2.1)
Short waiting time between services (lab. x-ray...)	78 (31.6)	143 (57.9)	9 (3.6)	16 (6.5)	1 (0.4)
Friendliness of staff	191 (45.5)	204 (48.6)	14 (3.3)	8 (1.9)	3 (0.7)
Attentiveness by the nursing staff (inpatient only *n* = 28)	9 (32.1)	12 (42.9)	4 (14.3)	2 (7.1)	1 (3.6)
Quality of food and other inpatient facilities (*n* = 28)	8 (28.6)	13 (46.4)	4 (14.3)	3 (10.7)	0

### Benefit and premium package of CBHI Scheme

Concerning the benefits package of the CBHI scheme, out of 420 clients, 280 (66.7%) of them were a very adequate benefit. Three hundred twenty-six (77.6%) clients perceived that the premium paid to the scheme is affordable. Two hundred ninety (69%) of clients were joined the CBHI scheme due of premium was low compared to out-of-pocket payment (OPP) while 218 (51.9%) were due to illness or injury that occurred frequently in their families (Table 3).

**Table 3:** Clients viewed about benefit and premium package of CBHI scheme implementation at Boru Meda hospital Feb.22-Mar 11/ 2019 (*n* = 420)

Variable		Frequency	Percent
CBHI benefit package	Very adequate	280	66.7
	Somewhat adequate	77	18.3
	Inadequate	60	14.3
	I don’t know	3	0.7
Premium	Affordable	326	77.6
affordability	Somewhat affordable	63	15
	Not affordable	31	7.4
Plan to renew membership	Yes	388	92.4
	No	32	7.6
Preference of hospital for future health care	Yes	337	80.2
	No	83	19.8

### Determining client satisfaction

The proportion of respondents who were satisfied with the health care service provided in this study was 80% at 95% CI (76.1–83.9%) computed from satisfaction measuring items.

### Factors associated with CBHI client satisfaction

In the first bivariate regression model; age (COR =2.39; 95% Cl: (1.02–5.59)) (*p* = 0.11),occupation, (COR =2.63; 95% Cl: (1.11–6.25)) (*p* = 0.16), marital status, (COR =0.54; 95% Cl: (0.32–0.90)) (*p* = 0.18) availability of drugs, (COR =0.12; 95% Cl: (0.07–.20)) (*p* = 0.03), waiting time to visit service provider in minutes, (COR =4.95; 95% Cl: (2.38–10.28)) (*p* = 0.04) plan to renew CBHI membership, (COR =6.27; 95% Cl: (2.97–13.23)) (*p* = 0.07), Prefer hospital for future health care, (COR =14.68; 95% Cl: (8.30–25.95)) (*p* = 0.08) and affordability of the scheme (COR =0.13; 95% Cl: (0.06–0.29)) (*p* = 0.14), were identified as factors associated with overall CBHI client’s satisfaction with *p*-value less than 20 included in multivariable logistic regressions.

In multivariable logistic regression, age, marital status, occupation and other significant variables in bi-variable logistic regression did not contribute to be independent predictors much for CBHI client satisfaction at (*p*-value < 0.05). However, availability of drugs and supply, waiting time to consult service provider within 30 min preceding consultation, prefer to the hospital for future health care need and plan to renew CBHI membership of the respondents were significantly associated with CBHI client’s satisfaction at (*p* < 0.05) and 95% Cl. Specifically, being the odds of availability of drugs none or partial, client’s satisfaction decline by 91% compared to full drugs available clients (AOR =0.09; 95% Cl: (0.04, 0.19)). Clients who had got service within 30 min were 3.16 times more likely satisfied than clients delayed for more than 60 min (AOR =3.16; 95% Cl: (1.19, 8.41)). Clients who had the plan to renew their CBHI membership were 4.96 times more likely satisfied compared to clients who did not plan to renew their CBHI membership (AOR =4.96; 95% Cl: (1.64, 15.02)). In addition, clients who preferred the hospital for future health care were 10.92 times more likely to be satisfied than clients who did not prefer the hospital for future health care (AOR =10.92; 95% Cl: (5.31, 22.45)) (Table 4).

**Table 4:** Bivaiate and multivariate analysis showing factors associated with CBHI client satisfaction at Boru Meda hospital, northeast, Ethiopia; Feb.22-Mar.11 /2019 (*N* = 420)

Explanatory variable	Client satisfaction		COR(95%)CI	AOR(95%)CI
	Satisfied N (%)	Dissatisfied N (%)		
Age (in years)				
< =18	64 (73.6)	23 (26.4)	1	1
19–29	31 (64.6)	17 (35.4)	0.65 (0.30–1.40)	0.72 (0.25–2.08)
30_39	60 (87)	9 (13)	2.39 (1.02–5.59)*	3.34 (1.00–10.36)
40_49	53 (85.5)	9 (14.5)	2.11 (0.9–4.96)	1.94 (0.59–6.41)
50_59	60 (83.3)	12 (16.7)	1.79 (0.82–3.92	2.86 (0.96–8.50)
> =60	68 (82.9)	14 (17.1)	1.74 (0.82–3.68)	1.51 (0.57–4.0)
Availability of drugs				
Fully	277 (90.2)	30 (9.8)	1	1
Partially& none	59 (52.2)	54 (47.8)	0.12 (0.07–.20)***	0.09 (0.04–0.19)***
Waiting time to visit service provider in (minutes)				
< 30	162 (91.5)	15 (8.5)	4.95(2.38–10.28)***	3.16 (1.19–8.41)*
31–60	126 (72.8)	47 (27.2)	1.22(0.67–2.25)	0.83 (0.38–1.95)
> 60	48 (68.6)	22 (31.4)	1	1
Prefer hospital for future health care				
Yes	304 (90.2)	33 (9.8)	14.68 (8.30–25.95)***	10.92 (5.31–22.45)***
No	32 (38.6)	51 (61.4)	1	1
Occupation				
Farmer	249 (83.3)	50 (16.7)	1	1
Unemployed	14 (60.9)	9 (39.1)	2.63 (1.11–6.25)*	0.99 (0.18–5.36)
Student	56 (77.8)	16 (22.2)	0.82 (0.257–0.263)	0.83 (0.17–3.97)
merchant	17 (65.4)	9 (34.6)	1.85 (0.69–4.94)	0.43 (0.09–2.04)
Plan to renew CBHI membership				
Yes	322 (83)	66 (17)	6.27 (2.97–13.23)***	4.96 (1.64–15.02)**
No	14 (43.8	18 (56.2)	1	1
Affordability of the scheme				
Affordable	274 (84.4)	51 (15.6)	1	1
Somewhat affordable	48 (76.2)	15 (23.2)	0.59 (0.30–1.13)	0.59 (0.30–1.13)
Not affordable	13 (41.9)	18 (58.1)	0.13 (0.06–0.29)*	0.64 (0.20–2.01)
Marital status				
Married	222 (82.8)	46 (17.2)	1	1
Single	86 (72.3)	33 (27.7)	0.54 (0.32–0.90)*	0.94 (0.21–4.1)
Divorced	12 (85.7)	2 (14.3)	1.10 (0.30–3.9)	3.77 (0.63–22.6)
Widowed	16 (84.2)	3 (14.8)	1. 20 (0.26–5.74)	1.34 (0.15–11.4)

(*) *P* -value < 0.05, (**) *P*- value < 0.01(***), *P*- value < 0.001. *AOR* Adjusted odd ratio, *COR* Crude odd ratio

## Discussion

According to the present study, clients who had got physician consultation within thirty minutes were 3.16 times more likely satisfied than clients who had delayed more than sixty minutes. This study supported the studies were conducted in the Nouna district of Burkina Faso, Tamil Nadu of South India, and Wolaita Sodo university teaching hospital [29, 31]. Therefore, long waiting time before physician consultation negatively affects client satisfaction.

In this finding, 92% of clients were having a plan to renew their CBHI membership which was higher than studies conducted in the Volta Region of Ghana 76%, and in Ethiopia 82% [32, 33]. The difference in the case of Ethiopia might be the variation in design and setup of a study while in Ghana might be socio-cultural and longtime experience in the scheme and health service provision. Clients with positive evaluation for the scheme and service provision influence their satisfaction [34, 35].

Being clients preferred to the hospital for future health care 10.92 times more likely satisfied than those who didn’t prefer the hospital for future health care needs. It might be due to clients was coming from health centers by referral so that relatively qualified health professionals and better diagnostic instruments were available in the hospital contributed their role. In addition, the perception of clients for the friendliness of staff and cleanliness of the hospital might have played a great role in the preference. This Study supported by a study conducted in the Nadowli District of Ghana indicated that health provider attitude and facility sanitation [36] positively influence client satisfaction.

### Limitation

Clients may stay relatively in a short time whereby they feel more satisfied immediately after their consultation than they do afterward. The use of quantitative techniques to explore perceptions might not have captured critical in-depth responses.

## Conclusion and recommendation

In general, the overall CBHI client satisfaction provided at Boru Meda hospital was low compared to the national CBHI evaluation. Full availability of prescribing drugs, clients renewed their CBHI membership, and preference of clients to use the hospital for future health care need positively associated with CBHI client satisfaction while the perception of waiting time before physician consultation negatively affected client’s satisfaction. Therefore, the hospital management members and service providers need to give attention to reduce waiting time preceding consultation, improve drug availability, and sustain the hospital preference by the hospital management members and service providers need to give attention to reduce waiting time preceding consultation, improve drug availability, and sustain the hospital preference by the client. Ministry of Health was also better to design and strengthen strategies sufficient quantity of essential drugs available in the public hospital, and improving public awareness regarding the concept of insurance. CBHI Agency should also follow and monitor the renewal period of CBHI members as per standard create a strategy to reimbursement for clients who did not get service in a contracted health institution. Further study will be proposed supporting with a qualitative study to determine satisfaction of CBHI scheme and health service provision in the study area and the region at large.

## Data Availability

*All* the datasets during and/or analyzed during the current study are available from the corresponding author on reasonable request.

## Abbreviations

APHI: Amhara Public Health Institute
CBHI: Community-based Health Insurance
FMOH: Federal Ministry of Health
OOP: out-of-pocket
SHI: social health insurance
